# Stellenwert der Computertomographie in der präoperativen Diagnostik der Otosklerose

**DOI:** 10.1007/s00106-022-01241-2

**Published:** 2022-11-03

**Authors:** Nadja Angela Stenz, Salman Hashmi, Dirk Lehnick, Thomas Treumann, Thomas Linder

**Affiliations:** 1 Klinik für Hals‑, Nasen- Ohren- und Gesichtschirurgie (HNO), Luzerner Kantonsspital, Spitalstraße, 6004 Luzern, Schweiz; 2 Gesundheitswissenschaften und Medizin, Universität Luzern, Luzern, Schweiz; 3 Klinik für Radiologie, Luzerner Kantonsspital, Luzern, Schweiz

**Keywords:** Audiometrie, Ohr, Bildgebung, Digitale Volumentomographie, Schwerhörigkeit, Audiometry, Ear, Imaging, Cone-beam CT, Hearing loss

## Abstract

**Hintergrund:**

Die Otosklerose ist ein häufig gesehenes Krankheitsbild in der HNO-ärztlichen Sprechstunde und führt durch einen Knochenumbau der otischen Kapsel zu einer progredienten Schwerhörigkeit. Die bildgebenden Methoden, insbesondere die Computertomographie (CT) und die digitale Volumentomographie (DVT), gewinnen in der Diagnose der Otosklerose zunehmend an Bedeutung.

**Ziel der Studie:**

Gibt es eine Korrelation zwischen dem Ausmaß der Otosklerose in der Bildgebung mittels CT oder DVT und der Hörminderung im Reintonaudiogramm?

**Material und Methoden:**

Aus bereits publizierten Einteilungskriterien zur Beurteilung von Otoskleroseherden wurde eine Klassifikation erarbeitet. Die präoperativen CT-Datensätze der im Zeitraum zwischen 2015 und 2019 operierten Patient*innen mit Otosklerose wurden von zwei unabhängigen HNO-Ärzt*innen evaluiert und klassifiziert. Die präoperativen Audiogramme wurden ausgewertet und mit den CT-Befunden verglichen.

**Resultate:**

Eingeschlossen wurden 168 Ohren von 156 Patient*innen mit intraoperativ bestätigter Otosklerose. Eine Korrelation zwischen der Ausdehnung der Otoskleroseherde bzw. dem errechneten Score und der Hörminderung (Luftleitung, Knochenleitung und Air-Bone-Gap) im Reintonaudiogramm konnte nicht nachgewiesen werden.

**Schlussfolgerung:**

Eine präoperative Diagnostik mittels CT ist nicht obligat. Die Bildgebung, bevorzugt mit einer DVT, kann jedoch zum Ausschluss weiterer Mittel- und Innenohrpathologien sowie zur Planung eines operativen Eingriffs im Kontext von Otoskopie und Audiometrie durchaus begründet sein. Eine Korrelation zur gemessenen Hörminderung bleibt weiterhin unklar und konnte in unserer Kohorte nicht nachgewiesen werden.

## Abstract (en)

**Background:**

Otosclerosis is an osteodystrophy of the otic capsule and presents with progressive conductive hearing loss. Imaging studies, especially computed tomography (CT) and cone-beam CT, have gained increased relevance in the diagnosis of otosclerosis.

**Objective:**

This study investigated whether there is a correlation between the extent of otosclerosis in high-resolution or cone-beam CT and hearing loss in pure-tone audiometry.

**Materials and methods:**

Based on an existing classification of otosclerotic foci, a classification was established. Preoperative CT scans of patients undergoing stapedotomy between 2015 and 2019 were evaluated and classified by two independent otorhinolaryngologists. The preoperative pure-tone audiograms were analysed and compared to the results of CT.

**Results:**

A total of 168 CT studies (i.e., 168 ears) in 156 patients with intraoperatively confirmed otosclerosis were included in our study. A correlation between the extent of the otosclerotic focus or the calculated scores and hearing loss in pure-tone audiometry (air conduction, bone conduction and air-bone-gap) could not be proven.

**Conclusion:**

Preoperative CT is not obligatory. However, preoperative imaging using CT or cone-beam CT can be helpful to confirm the diagnosis and exclude other middle or inner ear pathologies as well as in planning of the surgical procedure in the overall context of otoscopy and audiometry. A correlation with the degree of hearing impairment could not be demonstrated and remains unclear.

Die Otosklerose führt zu einem umschriebenen Knochenumbau der otischen Kapsel und ist ein häufiges Krankheitsbild in der HNO-ärztlichen Tätigkeit. Durch den Knochenumbau kommt es zu einer progredienten Schwerhörigkeit. Die Diagnose der Otosklerose konnte bisher nur intraoperativ oder histologisch gestellt werden. Mit dem technischen Fortschritt gewinnen nun zunehmend bildgebende Verfahren, insbesondere die Computertomographie (CT), an Bedeutung. Doch besteht auch eine Korrelation zwischen der in der CT sichtbaren Ausdehnung der Otosklerose und der Hörminderung im Reintonaudiogramm?

## Hintergrund

Die Otosklerose, eine in der HNO-Heilkunde verbreitete Diagnose, ist eine multifaktorielle Osteodystrophie, welche zu einem meist umschriebenen Umbau des Knochens der otischen Kapsel führt [2, 23, 24, 32]. Die Prävalenz der Erkrankung liegt histologisch bei rund 2,5 bis 10 %, ist klinisch jedoch deutlich geringer bei 0,3 bis 0,4 % und kann in 70 bis 85 % der Fälle bilateral auftreten [2, 9, 11, 23]. Die Otosklerose beginnt mit der aktiven, sog. otospongiotischen Phase, wobei Knochenbestandteile um die Blutgefäße resorbiert werden und sich perivaskuläre Räume bilden. In der Folge wird immaturer Knochen abgelagert, welcher während der inaktiven, otosklerotischen Phase der Erkrankung kalzifiziert und sich in lamellären Knochen umwandelt [23, 25, 32].

Das Krankheitsbild der Otosklerose führt zu einer progredienten Schwerhörigkeit. Audiometrisch lassen sich eine tieftonbetonte Schallleitungsschwerhörigkeit mit typischer Carhart-Senke bei 2 kHz, fehlende Stapediusreflexe sowie ein normales Tympanogramm bei gut belüftetem Mittelohr nachweisen. In der Ohrmikroskopie zeigt sich meist ein blander Befund, selten ist das Schwartze-Zeichen als eine Rötung im Bereich des Promontoriums durch einen hypervaskularisierten Otoskleroseherd zu erkennen. Oftmals wird über Tinnitus berichtet, während Schwindelsymptome zu atypischen Symptomen gezählt werden und bei Auftreten anderweitige Ursachen ausgeschlossen werden sollten. Die definitive Diagnose einer Otosklerose kann nur intraoperativ oder histologisch gestellt werden [5, 8, 11, 19, 21, 26, 32]. In den 1980er-Jahren konnte in ersten Studien gezeigt werden, dass die Otosklerose (antefenestral und cochleär) mittels CT nachweisbar ist [14, 29]. Mit weiteren Fortschritten in der Technologie gewinnen die bildgebenden Verfahren in der Diagnostik der Otosklerose an Bedeutung, insbesondere die Multidetektor-Computertomographie (MDCT) und zunehmend auch die digitale Volumentomographie (DVT, im Englischen als „cone-beam CT“ bezeichnet) [8, 12, 17, 23, 34]. Unter dem Begriff der Röntgenschnittbildverfahren werden die CT (von der Einzeilen- zur multiplanaren MDCT) und die DVT subsummiert. In der CT-Bildgebung stellt sich ein Otoskleroseherd in der aktiven Phase aufgrund der verminderten Knochendichte als hypodenser Herd der otischen Kapsel, als verdickte Stapesfußplatte, als verkleinerte Nische des runden oder ovalen Fensters oder als sog. Doppelringzeichen (Halo) um die Cochlea dar. Die Magnetresonanztomographie (MRT) hingegen spielt nur eine geringe Rolle in der Diagnostik der Otosklerose. So reichern die Otoskleroseherde nur während der aktiven Phase Kontrastmittel an, was nur auf dünnschichtigen T1-gewichteten oder FLAIR-Sequenzen sichtbar wird [11]. Die MRT mit Hydropssequenzen kann jedoch zur Diagnostik eines endolymphatischen Hydrops, insbesondere bei Innenohrsymptomatik (Perzeptionsschwerhörigkeit und Schwindel) sinnvoll sein, da eine Otosklerose mit entsprechender Symptomatik durchaus mit einem endolymphatischen Hydrops einhergehen kann [28, 33].

**Figure 1:**
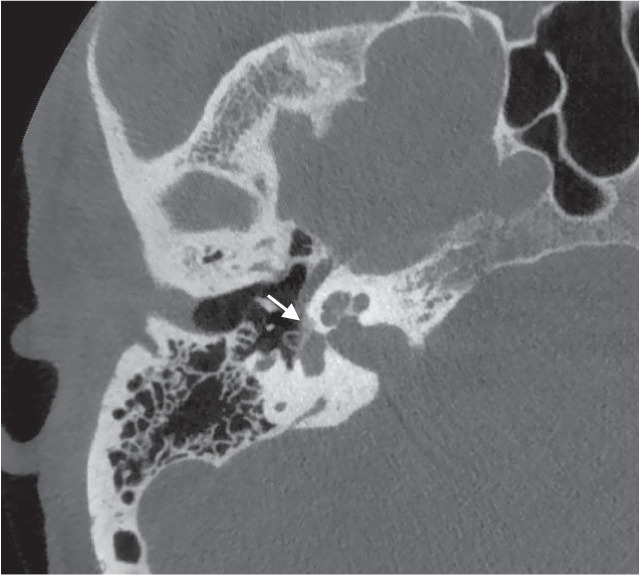

Grundsätzlich lässt sich die Otosklerose – je nach Lokalisation des Otoskleroseherds – in die häufigere antefenestrale und die seltener vorkommende retrofenestrale bzw. cochleäre Form einteilen (Abb. 1 und 2). Am häufigsten ist der hypodense Otoskleroseherd im Bereich der Fissula ante fenestram lokalisiert und kann sich dort bis zur kompletten Beteiligung der Stapesfußplatte und an die Cochlea ausdehnen [7, 11, 22, 34].

**Figure 2:**
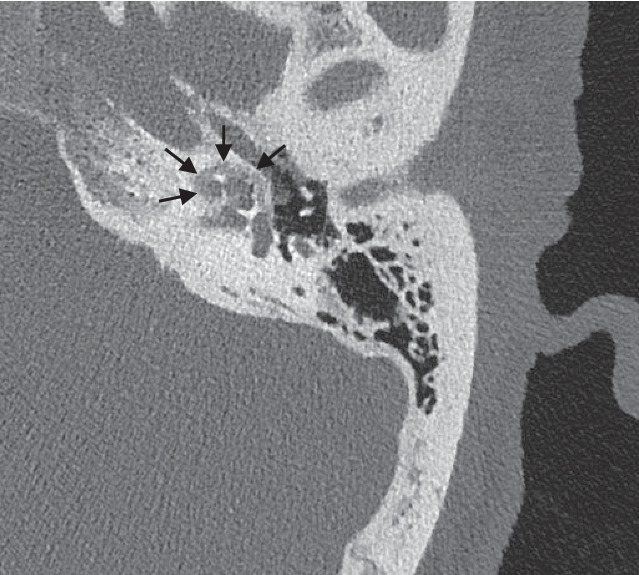

In der Literatur sind bisher mehrere Grading-Systeme zur Einteilung der Otosklerose in der CT beschrieben, jedoch konnte sich keines davon weitgehend durchsetzen. Einzig das Grading-System nach Symons und Fanning [16] wurde häufiger zitiert als andere, wobei eine sehr gute Übereinstimmung zwischen den Untersuchern sowie auch bei mehrmaliger Beurteilung durch einen einzelnen Untersucher gezeigt werden konnte [13]. Weitere Vorschläge zur Klassifikation der Otosklerose in der CT wurden unter anderem durch Veillon et al. [30] und Dudau et al. [4] beschrieben. Das Grading nach Veillon ist hauptsächlich in Frankreich verbreitet und teilt die Otosklerose nach Lokalisation in vier Typen ein. Dudau et al. beschrieben im Jahr 2017 eine Einteilung der Otosklerose, welche auch klinisch relevante Lokalisationen wie das runde Fenster oder die Beteiligung des cochleären Endosts einschließt.

### Studienziel

Das primäre Ziel unserer Studie war es, eine Korrelation zwischen Ausdehnung und/oder Lokalisation der Otoskleroseherde in der CT und der präoperativen Hörminderung (Luftleitung, Knochenleitung und Air-Bone-Gap) im Reintonaudiogramm nachzuweisen.

## Material und Methoden

### Patient*innen

Im Zeitraum zwischen Januar 2015 und Januar 2019 wurden an unserem Zentrum 287 Stapedotomien bei 272 Patient*innen im Alter zwischen 18 und 80 Jahren durchgeführt. Retrospektiv wurden hiervon 168 Ohren bzw. 156 Patient*innen aus der ENT-Statistics-Datenbank extrahiert und analysiert. Ausgeschlossen wurden die Fälle mit vorausgegangener Stapeschirurgie, intraoperativ nicht nachweisbarem Otoskleroseherd, Patient*innen ohne oder mit qualitativ ungenügender präoperativer computertomographischer Bildgebung sowie Patient*innen, welche die Weiterverwendung von Daten abgelehnt hatten.

### Bildgebung

Bei 147 der 168 Fällen lag eine MDCT mit Schichtdicken von 0,4 bis 1 mm vor, bei 21 Fällen eine DVT mit Schichtdicken von 0,12 bis 0,2 mm, vor. In allen Fällen wurden koronare und sagittale Rekonstruktionen angefertigt. Die Bildanalyse erfolgte an einer Picture-Archiving-and-Communication-System-Workstation (PACS-Workstation) an den Originalbildern, Rekonstruktionen und mittels live multiplanarer Reformation (MPR). Die Zeit zwischen der CT-Untersuchung und der Operation lag im Median bei 201 Tagen (Q1 131 Tage, Q3 452 Tage).

Bisherige Klassifikations- und Grading-Systeme der Otosklerose wurden adaptiert und das Ausmaß der Otosklerose in Bezug auf den vorderen Stapesschenkel, die Stapesfußplatte, das runde Fenster und die cochleäre Mitbeteiligung gradiert. Dadurch konnte ein Score für die jeweilige Lokalisation sowie auch in der Summe errechnet werden (Tab. 1). Die CT-Datensätze der 168 im untersuchten Zeitraum operierten Ohren wurden durch zwei Ärzt*innen (N. A. S. und S. H.) unabhängig voneinander klassifiziert und bei Uneinigkeit der erfahrenste Operateur beigezogen (T. L.) und so die Otosklerose klassifiziert.

**Table 1:** 

Score	A Antefenestral	F Fußplatte	C Cochleär	R Rundes Fenster
0	Nicht beteiligt	Nicht beteiligt	Nicht beteiligt	Nicht beteiligt
1	Beteiligt	≤ als 50 %	Basalwindung wird erreicht	Partiell beteiligt
2	–	> als 50 %	Großflächige Beteiligung der Basalwindung und der mittleren Windung	Obliteriert
3	–	Obliteriert	Otische Kapsel vollständig beteiligt (Halo)	–

### Audiometrie

Alle eingeschlossenen Patient*innen erhielten präoperativ ein Reintonaudiogramm. Die Luft- und Knochenleitung wurde für 0,5, 1, 2 und 4 kHz ermittelt. Der Air-Bone-Gap wurde aus der Differenz zwischen Luft- und Knochenleitung ermittelt. Der Mittelwert der Luft- und Knochenleitung wurde aus dem Durchschnitt der Hörschwellen bei 0,5, 1, 2 und 4 kHz ermittelt. Der mittlere Air-Bone-Gap errechnet sich aus der Differenz zwischen dem Mittelwert der Luft- und Knochenleitung. In unserer Analyse lag der Durchschnitt für die Luftleitung bei 51,7 dB (SD 16,4), für die Knochenleitung bei 25,6 dB (SD 11,2) und für den Air-Bone-Gap bei 26,1 dB (SD 9,6). Die zeitliche Latenz zwischen CT-Untersuchung und Audiometrie oder umgekehrt lag im Median bei 40 Tagen (Q1 7 Tage, Q3 161 Tage).

### Statistik

Demografische Daten der Studienpopulation wurden ebenso wie Parameter der CT-Klassifikation und der Audiogramme deskriptiv beschrieben. Kategoriale Variablen wurden in Form von Häufigkeitstabellen dargestellt. Quantitative Variablen wurden mittels deskriptiver Kennzahlen ausgewertet. Potenzielle Assoziationen zwischen den CT-Befunden bzw. den CT-Klassifikationen und der Hörminderung im Reintonaudiogramm wurde mithilfe von Kendalls Rangkorrelationskoffizienten tau‑b betrachtet. Die Güte der Übereinstimmung (Interrater Agreements) in der Beurteilung der CT wurden mittels Krippendorff‑α, gewichtet für ordinal skalierte Daten, beziffert. Die statistischen Analysen wurden unter Verwendung des Softwarepakets STATA (Version 15.1 oder höher, StataCorp, College Station, TX, USA) durchgeführt.

### Ethik

Unsere Studie wurde durch die Ethikkommission Nordwest- und Zentralschweiz bewilligt (Projekt-ID: 2019-00573).

## Ergebnisse

Die in dieser Arbeit analysierten 168 Ohren von 156 Patient*innen mit intraoperativ bestätigter Otosklerose wiesen ein Durchschnittsalter beim Eingriff von 49 Jahren (SD 11,4) auf. Über die Hälfte (56 %) der Fälle waren weiblich und in 51 % erfolgte die Intervention am linken Ohr. Sämtliche Patientencharakteristika sind in Tab. 2 aufgeführt.

**Table 2:** 

	MDCT (*n* = 147)		DVT (*n* = 21)		Total (*n* = 168)	
–	***n***	**%**	***n***	**%**	***n***	**%**
Männlich	65	44	9	43	74	44
Weiblich	82	56	12	57	94	56
*Seite*						
Rechts	73	50	9	43	82	49
Links	74	50	12	57	86	51
–	**Median**	**Q1, Q3**	**Median**	**Q1, Q3**	**Median**	**Q1, Q3**
Alter, Jahre	49	42, 56	50	38, 61	49	41, 56
LL, dB (0,5–4 kHz)	50	40, 60	51	44, 61	50	41, 60
KL dB (0,5–4 kHz)	24	18, 31	25	20, 34	24	18, 31
ABG, dB (0,5–4 kHz)	25	19, 33	26	23, 33	25	19, 33

*MDCT* Multidetektor-Computertomographie, *DVT* digitale Volumentomographie, *LL* Luftleitung, *KL* Knochenleitung, *ABG* Air-Bone-Gap

In 98 % der Fälle wurde die intraoperativ bestätigte Otosklerose in der präoperativen MDCT oder DVT detektiert. Nach der ersten, unabhängig durchgeführten Klassifikation der Bilddatensätze durch zwei Ärzt*innen zeigte sich sowohl in der MDCT als auch in der DVT eine sehr hohe Übereinstimmung der Klassifikation zwischen beiden Beurteilern. Im totalen Score wurde ein Krippendorff‑α von 0,91 erreicht (Tab. 3). Bei der MDCT ergab sich im Total Score ein Krippendorff‑α von 0,90; in den 21 Fällen der DVT-Gruppe war die Übereinstimmung sogar nahezu ideal (Krippendorff-α = 0,95).

**Table 3:** 

	MDCT (*n* = 147)					DVT (*n* = 21)					Total (*n* = 168)				
	A	F	C	R	Total Score	A	F	C	R	Total Score	A	F	C	R	Total Score
Prozentuale Übereinstimmung (ungewichtet) (95%-KI)	0,93 (0,88; 0,97)	0,88 (0,83; 0,94)	0,93 (0,89; 0,97)	0,99 (0,97; 1,0)	0,80 (0,73; 0,86)	1,0 (0,84; 1,0)	0,90 (0,77; 1,0)	1,0 (0,84; 1,0)	1,0 (0,84; 1,0)	0,90 (0,77; 1,0)	0,93 (0,90; 0,97)	0,89 (0,84; 0,94)	0,94 (0,90; 0,98)	0,99 (0,97; 1,0)	0,81 (0,75; 0,87)
Krippendorff‑α (ordinal gewichtet) (95%-KI)	0,32 (0,01; 0,62)	0,86 (0,79; 0,93)	0,93 (0,88; 0,97)	0,94 (0,86; 1,0)	0,90 (0,86; 0,94)	1,0 n. b.	0,86 (0,65; 1,0)	1,0 n. b.	1,0 n. b.	0,95 (0,88; 1,0)	0,39 (0,10; 0,68)	0,86 (0,80; 0,92)	0,94 (0,90; 0,98)	0,95 (0,88; 1,0)	0,91 (0,87; 0,94)

Krippendorff-α < 0,2 schwache, 0,21–0,4 angemessene, 0,41–0,6 moderate, 0,61–0,8 gute, 0,81–1,0 sehr gute/fast perfekte Übereinstimmung

*A* antefenestral, *F* Fußplatte, *C* cochleär, *R* rundes Fenster, *MDCT* Multidetektor-Computertomographie, *DVT* digitale Volumentomographie, *n. b.* nicht beurteilbar

Ein relativ niedriges Krippendorff‑α von 0,32 ergab sich, trotz gleichzeitig hoher prozentualer Übereinstimmung, lediglich für die Klassifikation der antefenestralen Otosklerose bei der MDCT.

Bei 163 Scans konnte eine antefenestrale Otosklerose nachgewiesen werden, wohingegen eine cochleäre Otosklerose in 40 Fällen und eine Beteiligung des runden Fensters in 13 Fällen vorlag. Die Tab. 4 und 5 zeigen den Zusammenhang zwischen den Klassifikationen und der durchschnittlichen Luft- und Knochenleitung sowie dem Air-Bone-Gap.

**Table 4:** 

	A Antefenestral				F Fußplatte				C Cochleär				R Rundes Fenster			
Score	*n*	%	Median ABG (dB) (Q1, Q3)	Median KL (dB) (Q1, Q3)	*n*	%	Median ABG (dB) (Q1, Q3)	Median KL (dB) (Q1, Q3)	*n*	%	Median ABG (dB) (Q1, Q3)	Median KL (dB) (Q1, Q3)	*n*	%	Median ABG (dB) (Q1, Q3)	Median KL (dB) (Q1, Q3)
0	5	3	33,8 (23,8; 33,8)	23,8 (22,5; 25)	39	23	25 (18,8; 32,5)	26,3 (18,8; 36,3)	128	76	25 (18,8; 33,8)	22,5 (17,5; 31,3)	155	92	25 (18,8; 32,5)	23,8 (17,5; 31,3)
1	163	97	25 (28,8; 32,5)	23,8 (27,5; 31,3)	97	58	25 (18,8; 32,5)	22,5 (17,5; 31,3)	21	12	22,5 (21,3; 26,3)	27,5 (15; 31,3)	9	5	27,5 (22,5; 33,8)	25 (21,3; 31,3)
2	–	–	–	–	29	17	25 (20; 30)	25 (18,8; 28,8)	11	7	26,3 (22,5; 35)	25 (18,8; 30)	4	4	32,5 (25,6; 36,3)	23,8 (16,3; 42,5)
3	–	–	–	–	3	2	36,3 (32,5; 48,8)	18,8 (8,8; 31,3)	8	5	26,3 (23,1; 28,1)	22,5 (18,1; 41,9)	–	–	–	–
Total	168	100	25 (19,4; 32,5)	23,8 (28,1; 31,3)	168	100	25 (19,4; 32,5)	23,8 (18,1; 31,3)	168	100	25 (19,4; 32,5)	23,8 (18,1; 31,3)	168	100	25 (19,4; 32,5)	23,8 (18,1; 31,3)

*ABG* Air-Bone-Gap, *KL* Knochenleitung

**Table 5:** 

Audiogramm	A Antefenestral	F Fußplatte	C Cochleär	R Rundes Fenster	Total Score
LL	−0,005 (*p* = 0,94)	−0,031 (*p* = 0,61)	0,025 (*p* = 0,69)	0,057 (*p* = 0,37)	−0,006 (*p* = 0,92)
KL	0,005 (*p* = 0,94)	−0,080 (*p* = 0,19)	0,037 (*p* = 0,55)	0,030 (*p* = 0,64)	−0,049 (*p* = 0,41)
ABG	−0,034 (*p* = 0,60)	0,039 (*p* = 0,53)	0,003 (*p* = 0,96)	0,081 (*p* = 0,21)	0,041 (*p* = 0,49)

*LL* Luftleitung, *KL* Knochenleitung, *ABG* Air-Bone-Gap

**Figure 3:**
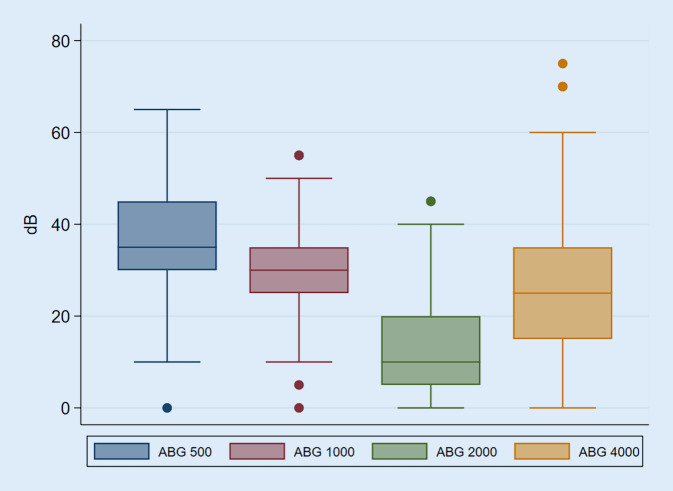

Die Audiogramme zeigten verlässlich die zu erwartende Carhart-Senke bei 2 kHz sowie die typische Konfiguration der tieftonbetonten Schallleitungsschwerhörigkeit der Otosklerose (Abb. 3 und 4). Im Vergleich zwischen den errechneten totalen Scores und der Hörminderung im Reintonaudiogramm – sowohl Luft-, als auch Knochenleitung und dem Air-Bone-Gap – konnte keine signifikante Korrelation gefunden werden. Wie in Tab. 5 dargestellt, zeigte sich auch zwischen dem Score der Beteiligung antefenestral, der Fußplatte und des runden Fensters und der Hörminderung im Reintonaudiogramm keine Korrelation. Auch zwischen der Beteiligung der Cochlea und der Hörminderung – insbesondere der Innenohrleistung – ergab sich keine signifikante Korrelation (Tab. 5). Dies konnte sowohl über alle CT-Datensätze als auch aufgeschlüsselt nach DVT und MDCT-Untersuchung gezeigt werden.

**Figure 4:**
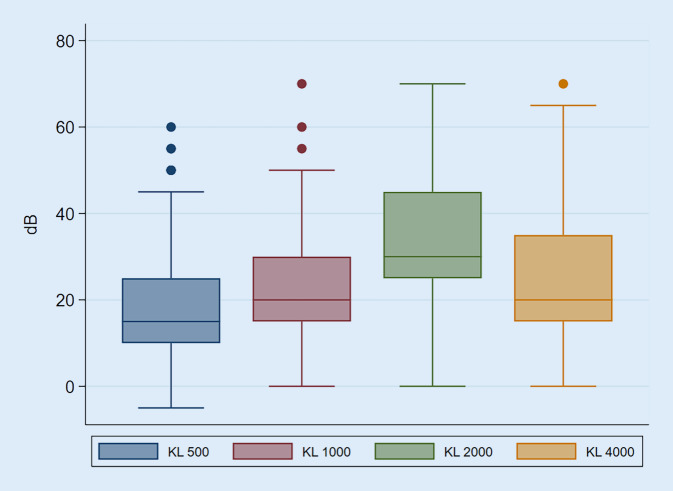

## Diskussion

Es gilt heute als gesichert, dass mittels Röntgenschnittbildverfahren die Otosklerose in einem hohen Prozentsatz nachgewiesen werden kann. Die meist antefenestral lokalisierten Otosklerosefoci konnten in verschiedenen Publikationen in 70 bis 95 % sichtbar gemacht werden [12, 17, 21, 27, 31], während die Detektionsrate in unserer Arbeit bei 98 % lag. In älteren Studien [21] lag die Nachweisrate der Otosklerose in der CT bei lediglich 70 %. Diese unterschiedlichen Nachweisraten begründen sich in der unterschiedlichen CT-Technologie, der Schichtdicke (wobei dünnste 0,1-mm-Bilder wegen des hohen Bildrauschens nicht optimal ausfallen), der Kollimation, dem Pitch, Zoom und auch den Auswertemöglichkeiten an den Workstations. Dagegen konnte sowohl mittels histologischen Untersuchungen als auch im Vergleich mit dem intraoperativen Befund gezeigt werden, dass die CT-Bildgebung sehr sensitiv und spezifisch für die Diagnose einer Otosklerose ist [12], einzig die Beteiligung des Endosts konnte nicht klar beurteilt werden [23].

Bei typischer Anamnese, regelrechter Otoskopie (evtl. Schwarze-Zeichen) und passenden audiometrischen Befunden hinweisend auf eine wahrscheinliche Otosklerose erachten wir die Durchführung einer Röntgenschnittbildgebung vor Durchführung einer Stapedotomie als nicht obligat. Bei einem Revisionseingriff oder Zweifel an der Diagnose der Otosklerose, wie beispielsweise bei kombinierter Schwerhörigkeit, Perzeptionsschwerhörigkeit oder pancochleärer Schallleitungsschwerhörigkeit, fluktuierender Hörminderung, Anamnese von rezidivierenden Mittelohrinfekten, zusätzlicher vestibulärer Symptomatik oder jungem Alter der Patient*innen (< 30 Jahren), können mittels präoperativer Bildgebung Hinweise für mögliche anderweitige Mittelohrpathologien (z. B. Dislokation/Fixation der Ossikelkette, Tympanosklerose) oder Pathologien des Felsenbeins, wie eine Dehiszenz des superioren Bogengangs, Osteogenesis imperfecta, Morbus Paget als wichtige Differenzialdiagnosen einer Otosklerose, erkannt werden. Die sorgfältige Analyse einer präoperativen Bildgebung kann ebenfalls mögliche intraoperative Komplikationen rechtzeitig erkennen. Dazu gehören Innenohrmalformationen, wie ein „large vestibular aqueduct“ oder eine „X-linked mixed deafness“ mit einem intraoperativen Risiko eines perilymphatischen Gushers. Aber auch ein möglicher überhängender N. facialis im tympanalen Segment oder die Persistenz einer A. stapedia können bereits präoperativ erkannt werden [18, 35].

Die Fortschritte der MDCT-Technik mit dünneren Schichtdicken und der Einsatz der DVT waren über den Studienzeitraum erkennbar, so auch in der gering besseren, jedoch nicht signifikanten Übereinstimmung der Klassifikation zwischen verschiedenen Betrachtern bei der DVT (Krippendorff‑α 0,90 (95%-KI 0,86; 0,94)) verglichen mit der MDCT (Krippendorff‑α 0,95; 95%-KI 0,88; 1,0). Dies ist aus unserer Sicht auf die bessere Auflösung und geringeren Schichtdicken der DVT zurückzuführen mit entsprechend besserer Auflösung in der Z‑Achse. Einzig bei der Klassifikation der antefenestralen Otosklerose ergab sich ein relativ niedriges Krippendorff‑α von 0,39; bei jedoch hoher prozentualer Übereinstimmung der Klassifikation von 93 % (Tab. 3). Dies erklärt sich aus dem Umstand, dass es nur fünf Fälle ohne Beteiligung der antefenestralen Region gab und sich die Ärzt*innen in der Klassifikation dieser seltenen Fälle teilweise uneinig waren.

Die geringe Strahlendosis wird bei der DVT ebenfalls als vorteilhaft hervorgehoben [3]. Gerade in der Mittelohrchirurgie entscheidend ist jedoch die hohe Auflösung bezüglich der Knochenstrukturen, wie beispielsweise der Ossikelkette, des Verlaufs des N. facialis oder der Stapesfußplatte [6].

Bereits im Jahr 1985 [25] wurde in einer histologischen Studie die Beziehung zwischen der Lokalisation bzw. Ausdehnung der Otosklerose und dem Tonaudiogramm untersucht, wobei keine Korrelation zwischen der Größe der Otosklerose sowie auch der Beteiligung des Endosts und der Hörminderung gefunden werden konnte. Bezüglich der Korrelation zwischen der Otosklerose in der Bildgebung und der Hörminderung im Tonaudiogramm existieren kontroverse Meinungen. So konnten in Studien aus dem Jahr 2010 aus Korea [20] und einer Arbeit aus dem Jahr 2014 aus Ägypten [1] keine Korrelation zwischen der Größe des otosklerotischen Fokus und der Knochenleitung sowie des Air-Bone-Gap im Tonaudiogramm aufgezeigt werden. Auch in unseren Daten zeigt sich keine Korrelation zwischen der Größe des Otosklerosefokus und der Knochenleitung sowie dem Air-Bone-Gap bzw. Luftleitung. Hingegen zeigten Shin et al. [27] in Frankreich, dass die Knochenleitungsschwelle bei Mitbeteiligung des Endosts höher lag, als wenn keine Beteiligung des Endosts vorlag. Marx et al. [17] berichteten, dass sowohl die Luft- als auch Knochenleitungsschwelle schlechter ausfielen im Fall einer extensiven Otosklerose im CT-Befund verglichen mit einer lediglich antefenestral lokalisierten Otosklerose. Naumann et al. [21] fanden zwar eine Korrelation zwischen der Größe des Otosklerosefokus bezogen auf den Air-Bone-Gap, jedoch nicht auf die Innenohrleistung. Bezüglich der Lokalisation bzw. Ausdehnung der Otosklerose in die Rund-Fenster-Nische konnten unsere Daten keine Korrelation zur Hörminderung (sowohl Knochen- als auch Luftleitung sowie Air-Bone-Gap) zeigen. Dies steht im Kontrast zu einer Arbeit aus dem Jahr 2011, in welcher gezeigt werden konnte, dass bei Beteiligung der gesamten Rund-Fenster-Nische sowie bei kompletter Obliteration sowohl eine schlechtere Luft- als auch Knochenleitung und ein größerer Air-Bone-Gap gefunden werden konnte [15].

Auch wurde die Beziehung zwischen der Densität der Otosklerose in Hounsfield-Einheiten in der CT-Bildgebung mit einer Auflösung von 0,5 mm und der Hörleistung untersucht. Hierbei konnten Kawase et al. [10] eine Korrelation zwischen den Hounsfield-Einheiten der antefenestralen Otosklerose und der Hörschwelle bei 0,5 und 1 kHz zeigen. Abdel-Ghany et al. [1] konnten hingegen zeigen, dass keine Korrelation zwischen der Densität der Otoskleroseherde und der Hörminderung vorliegt.

Als Limitationen unserer Studie sehen wir einerseits die unterschiedlichen CT-Datensätze, welche mit unterschiedlichen Geräten und teilweise an unterschiedlichen Standorten durchgeführt wurden. Außerdem wurden nur Fälle mit der intraoperativ bestätigten Diagnose einer Otosklerose analysiert, dies führt möglicherweise zu einem Selektionsbias. Andererseits fiel auch die Uneinheitlichkeit der präoperativen Abklärungen auf, welche sich durch die große Streubreite der Zeiträume zwischen Audiogramm und CT äußern. Optimal wäre ein einheitlicher kurzer Zeitraum zwischen CT-Untersuchung und Audiometrie, um so eine allfällige Progression der Otoskleroseherde in der CT oder der Hörminderung im Audiogramm zu vermeiden. Allerdings zeigen unsere persönlichen Erfahrungen bei wiederholten CT-Untersuchungen über mehrere Jahre kaum nachweisbare Veränderungen.

## Fazit für die Praxis

- Bei vermuteter Otosklerose kann die CT- oder DVT-Untersuchung einen Otoskleroseherd in den allermeisten Fällen nachweisen.
- Dabei sollte die Schichtdicke möglichst dünn (> 0,1 mm und < 0,6 mm) gewählt oder bevorzugt eine DVT angefordert werden.
- Mehrere Publikationen, inklusive der vorliegenden Studie, konnten jedoch keine Korrelation zwischen der Hörminderung im Reintonaudiogramm und der radiologischen Ausdehnung der Otosklerose nachweisen.
- Bei hohem klinischem und audiologischem Verdacht auf eine Otosklerose ist eine MDCT oder eine DVT zwar nicht obligat, die immer bessere Auflösung der neusten Gerätetechnologien erlaubt es jedoch, neben der Bestätigung der Diagnose einer Otosklerose relevante Differenzialdiagnosen auszuschließen (z. B. Malformation der Ossikelkette, Ossifikationen des Hammerligaments, Dehiszenz des superioren Bogengangs) oder wichtige Nebenbefunde zur Operationsplanung einer Stapedotomie (z. B. Präsenz einer A. stapedia, Position des N. facialis, erweiterte Innenohrräume) vorausschauend nachzuweisen.
- In seltenen Fällen kann auch die Bildgebung mittels MRT zur begleitenden Diagnose eines endolymphatischen Hydrops hilfreich sein.
